# Characterization of a novel fast-growing zebrafish: a new approach to growth hormone transgenesis

**DOI:** 10.3389/fendo.2024.1369043

**Published:** 2024-04-02

**Authors:** Noam Cohen-Rothschild, Naama Mizrahi, Berta Levavi-Sivan

**Affiliations:** Department of Animal Sciences, The Robert H. Smith Faculty of Agriculture, Food and Environment, The Hebrew University of Jerusalem, Rehovot, Israel

**Keywords:** LH, transgenic fish, GH, pituitary, growth

## Abstract

The manipulation of the somatotropic axis, governing growth, has been a focus of numerous transgenic approaches aimed at developing fast-growing fish for research, medicine and aquaculture purposes. However, the excessively high growth hormone (GH) levels in these transgenic fish often result in deformities that impact both fish health and consumer acceptance. In an effort to mitigate these issues and synchronize exogenous GH expression with reproductive processes, we employed a novel transgenic construct driven by a tilapia luteinizing hormone (LH) promoter. This approach was anticipated to induce more localized and lower exogenous GH secretion. In this study, we characterized the growth and reproduction of these transgenic LHp-GH zebrafish using hormonal and physiological parameters. Our findings reveal that LHp-GH fish exhibited accelerated growth in both length and weight, along with a lower feed conversion ratio, indicating more efficient feed utilization, all while maintaining unchanged body proportions. These fish demonstrated higher expression levels of LH and GH in the pituitary and elevated IGF-1 levels in the liver compared to wild-type fish. An examination of reproductive function in LHp-GH fish unveiled lower pituitary LH and FSH contents, smaller follicle diameter in female gonads, and reduced relative fecundity. However, in transgenic males, neither the distribution of spermatogenesis stages nor sperm concentrations differed significantly between the fish lines. These results suggest that coupling exogenous GH expression with endogenous LH expression in females directs resource investment toward somatic growth at the expense of reproductive processes. Consequently, we conclude that incorporating GH under the LH promoter represents a suitable construct for the genetic engineering of commercial fish species, providing accelerated growth while preserving body proportions.

## Introduction

The fundamental processes of energy homeostasis, growth, and reproduction, which are essential for fitness and survival, are intimately related. Growth is a polygenetic trait influenced by several physiological pathways connected to energy usage, metabolism, and reproduction (1). The somatotropic axis, which regulates growth in all vertebrates, has, among others, two main components: growth hormone (GH) and insulin-like growth factor-1 (IGF-1). However, the control of the release of GH from the pituitary is complicated and combines growth hormone-releasing hormone (GHRH), gonadotropin-releasing hormone (GnRH), and dopamine, which upregulate GH expression, and somatostatin, which inhibits GH expression (2–6). GH induces elevated free fatty acid levels, hyperplastic muscle growth, and stimulates appetite (7). In addition, GH is responsible for localized secretion of IGF-1 in fat, muscle, and bone tissues, as well as for IGF-1 secretion from the liver into the bloodstream. In fish, IGF-1 affects muscle growth, protein synthesis, and myoblast proliferation (1). In mammals, it has been shown to directly regulate bone growth and density (8). IGF-1 also mediates a negative feedback loop within the somatotropic axis (7).

As mentioned, somatic growth is tightly coupled with reproductive cycles. The hypothalamic-pituitary-gonadal axis produces the hormonal network that regulates reproductive function. GnRH, which is released from the hypothalamus, stimulates the synthesis and release of the gonadotropins, luteinizing hormone (LH) and follicle-stimulating hormone (FSH) from the anterior pituitary. These act individually or cooperatively on the gonads to regulate steroidogenesis and gametogenesis. Whereas FSH stimulates the growth of ovarian follicles in females and spermatogenesis in males, LH induces ovulation in females and androgen secretion in males (9, 10).

Unlike mammals, whose growth stops when they reach sexual maturity, fish grow throughout their life span (11), switching from somatic growth to reproduction and vice versa. The coordination of these two processes requires communication and reciprocal regulation between the somatotropic and hypothalamic-pituitary-gonadal axes (12). For example, GH is regarded as a secondary reproduction hormone (10). In female catfish, GH injections stimulate ovarian growth and development (13), and in rainbow trout, plasma GH levels are associated with gonadal maturation (14). Furthermore, in killifish, GH stimulates E2 synthesis by ovarian tissue (15). IGF-1 has been shown to induce LH synthesis and release *in vitro* by pituitary cells of juvenile female eels (16) and to increase FSH content and GnRH-stimulated FSH release from juvenile coho salmon pituitary *in vitro* (17). Moreover, IGF-I increased FSH and LH sensitivity to GnRH in rainbow trout *in vitro* (18). In goldfish, E2 stimulates GH secretion throughout the reproductive cycle of female goldfish. Furthermore, GnRH3 stimulates GH release in a teleost (19, 20). These interactions constitute the crosstalk between the two axes and could control the energy shifts between somatic growth and reproduction.

The somatotropic axis is a perfect candidate for manipulation through transgenesis to produce fast-growing organisms for research and agriculture, particularly aquaculture. Fish provide a large portion of the global protein intake by humans (21). Given the constant growth in human population and the depletion of natural fish stocks worldwide, supply increasingly relies on cultured fish. Transgenic lines of fast-growing fish have been created from various teleosts, such as coho salmon (22) and domestic and wild rainbow trout (23), expressing the GH-I gene of sockeye salmon driven by the metallothionein-B promoter from the same species. Other attempts included driving GH expression in Mutiara catfish by cytomegalovirus (24), an “all body” GH expression driven by a β-actin gene promoter in common carp (25), GH expressed under the regulation of medaka β-actin in tilapia (26), and zebrafish containing a carp β-actin promoter driving the expression of the marine silverside fish GH (27).

The strategy of driving GH using an “all body”, highly active promoter has produced very fast-growing fish that suffer from health-related problems and abnormal morphological changes (23, 28–30). To solve these issues, we employed a different approach to GH transgenesis in this study that reduces exogenous GH expression by using a less active and, more specifically, localized promoter. Moreover, exogenous GH expression was induced by LH promoter to couple between somatic growth and reproductive processes. In addition to possibly redirecting metabolic resources from sexual maturation and reproduction to somatic growth and thereby ensuring uninterrupted growth, we postulated that this approach would also provide a more conventional transport system for the hormone to reach its target tissues. We used zebrafish, an established model organism with a fully sequenced genome (31, 32), which is widely used to study aging (33), immune system (34), endocrine models (35), heart arrhythmia (36), and cancer research (37), as well as to search for neuropeptides regulating reproduction (38). To characterize this novel line of transgenic zebrafish, we compared hormonal and physiological parameters of growth and reproduction to those of wild-type fish. Our results indicate that the new construct drives increased somatic growth while suppressing reproductive functions without altering body proportions.

## Methods

### Transgenic construct

For this study, we generated a new transgenic line of zebrafish, Tg(LHp : GH, cmlc2:EGFP), by using Tol2kit (39) as described in Invitrogen Multisite Gateway Manual. Tilapia LH promoter (NC_031978.2) (40) and tilapia GH (NC_031969.2) DNA fragments were amplified by PCR. The LH promoter was inserted into pDONRP4-P1R, and the tilapia GH was cloned into pDONR221, through BP recombination. The 5′-entry clone (p5E), middle-entry clone (pME), and a 3′-entry clone (p3E), which contained a polyA sequence, were then recombined through an LR reaction into the expression vector pDestTol2CG, which finally carries the tiLH promoter, tilapia GH, GFP heart marker and tol2-recognition sequences.

In order to perform the transgenesis, eggs were promptly collected post-spawning and were injected with a mixture of expression plasmid, transposase mRNA, phenol red, and DEPC-treated molecular biology water. After hatching, larvas were screened for EGFP fluorescent marker in the heart using a fluorescence stereo microscope. EGFP-positive embryos were grown and mated as possible founders. Heterozygous mutants were generated by crossing the F0 pLH-GH with WT fish. Then, verified F1 male and female fish were crossed to generate homozygous transgenic fish.

### Fish maintenance, measurements and sample collection

Transgenic zebrafish were maintained in a stand-alone unit with central filtration and heating (28 ± 1°C). The fish were fed in excess (food weight for day >3% averaged body weight) twice daily with commercial feed (Aquazone Ltd., Tzofit, Israel). Fish from the F1 generation of both sexes were placed in the same aquarium for breeding. Fertilized eggs were collected the next morning and incubated for 4 days. Larvae were screened and separated into transgenic (LHp-GH) and wild-type (WT), then transferred into brackish (5 ppt) water in stand-alone tanks and fed with rotifers (*Brachionus plicatilis*) until 14 days post-fertilization (dpf). Then, larvae were transferred to fresh water and fed with 1 ml of artemia until 28 dpf, after which the fish were fed once a day with 300 mg of commercial feed per tank. Overall, fish from the F2 generation were grown for 3 months to reach sexual maturation in two experimental cycles.

Fish length and weight were measured weekly. Weekly weight values were used to calculate the feed conversion ratio [FCR; feed intake (mg)/weight gain (mg)] and specific growth rate (SGR); % day^-1^ ln (W)- ln (W_0_)/Δ*T* X100, where W is the weight at the end of the experimental period (in mg), W_0_ is the weight at the beginning of the experimental period (in mg), and ΔT is the experimental period (in days). At the age of 3 months, the fish were anesthetized and dissected. The gonads, liver, and visceral fat tissue were weighed in order to calculate the gonadosomatic index (GSI), hepatosomatic index (HSI), and visceral fat somatic index (VFSI), respectively. Somatic indices were calculated as the percentage of the tissue weight from the body weight (BW).

Brains and livers were collected in Eppendorf Safe-Lock tubes and placed in liquid nitrogen for subsequent total RNA extraction. From each fish, one gonad was collected for total RNA extraction, and the other was fixed in Bouin’s fluid for histology, as described previously in Biran et al. (2008) (38). Pituitaries were collected from WT and LHp-GH fish, and half were prepared for total RNA extraction, and the other half were placed in DDW and kept at -20°C for subsequent enzyme-linked immunosorbent assays (ELISA).

All experimental procedures followed the Animal Care and Use Guidelines of the Hebrew University and were approved by the local Administrative Panel on Laboratory Animal Care.

### Immunohistochemistry of zebrafish pituitaries

Immunofluorescent staining for tilapia GH and carp LHβ was generally performed as outlined previously (41). Briefly, tissue samples were fixed in 4% paraformaldehyde, immersed in a cryoprotecting agent (30% sucrose), and frozen in an OCT embedding compound. Samples were sectioned at a thickness of 15 μm on a cryostat and collected on Superfrost slides. The sections were blocked with 5% normal goat serum (NGS) for 1 hour to reduce non-specific reactions and then incubated with rabbit anti-tiGH (diluted 1:500) (42) or rabbit anti-carp LHβ (diluted 1:200) (43) for 16 hours at 4°C. Antibodies were diluted in PBS with 1% BSA and 0.3% Triton X-100. The slides were rinsed three times with PBS for 5 minutes and were incubated for 2 hours at room temperature with goat anti-rabbit antibodies conjugated to Alexa488 or Alexa495 fluorophore.

For double-labeled staining, sections were blocked again with NGS (1X 5% PBS, 0.3% NGS in Triton X-100) for 1 hour at room temperature to saturate the open binding sites on the first secondary antibody with IgG. The slides were rinsed three times with PBS for 5 minutes and then incubated for 1 hour with an excess of unconjugated Fab Goat Anti-Rabbit IgG (Enco) diluted at 1:65 in an antibody dilution buffer. The slides were rinsed again three times, and the second primary antibody was applied (anti-rtiGH or anti-cLHβ) for 16 hours at 4°C. After an additional wash, the slides were incubated with the second secondary antibody for 2 hours at 25°C. After washing, slides were mounted with anti-fade solution (2% propyl gallate, 75% glycerol, in PBS) and imaged with confocal microscopy. All staining processes were performed in the dark.

### ELISA for gonadotropins and GH

A specific and homologous ELISA for carp LH (cLH), carp FSH (cFSH) and tilapia GH (tiGH) measurements had previously been established in our lab (42, 44–46) The binding of antibodies for cLH and cFSH to zebrafish LH and FSH were validated via immunohistochemical staining (46).

The competitive ELISA for LH and FSH was performed by using primary antibodies against recombinant cFSHβ at a 1:50,000 dilution, recombinant LHβ at a 1:14,000 dilution and recombinant tiGH at a 1:15,000 dilution (45, 46) and (43, 45, 46), respectively. Recombinant cFSHβα, cLHβα and tiGH were adopted as a standard, while the wells were coated with cFSHβ (50 ng/ml), cLHβ (100 ng/ml) and tiGH (25 ng/ml). A second antibody, GAR-HRP (Zotal Israel), was added at a 1:10,000 dilution, and the presence of enzyme complexes was visualized by the addition of 100 μl/well of a 3, 3’, 5, 5’-tetramethylbenzidine peroxidase substrate (KPL, Zotal, Israel). The reaction was carried out in complete darkness at room temperature, and absorbance was recorded at 450 nm after 15 minutes using a Sunrise ELISA reader (Tecan, Switzerland). The sensitivity for the measurements was 15.84 pg/ml for LH, 0.24 pg/ml for FSH and 35.0 pg/ml for GH. The inter-assay coefficient of variation (CV) was 14.8, 12.5, and 13%, while intra-assay CV was 7.2, 8, and 8% for LH, FSH and GH, respectively. The parallelism between the standard curve and serial dilutions of zebrafish pituitary extract was detected for both FSH and LH ELISAs (**Supplementary Figure 1**).

LHβ, FSHβ and tiGH content in the pituitary were measured by ELISA as described above. Pituitary extracts were prepared in 0.1% PBS. The pituitary extracts were diluted for LH, FSH and GH measurements at 1:25. Each sample was run in duplicate.

### RNA extraction and real-time PCR

RNA extraction and real-time PCR were performed generally according to (38, 45). Briefly, total RNA was extracted from the pituitaries using Trizol reagent (GIBCO, USA) according to the manufacturer’s protocol. cDNA (8 ng/µl RNA for pituitary; 40 ng/µl RNA for brain and liver). was prepared with the Verso cDNA Synthesis Kit (Thermo Fisher Scientific, Waltham, MA, USA) according to the manufacturer’s instructions. mRNA levels were normalized by the comparative threshold cycle method against the housekeeping gene elongation factor 1α (ef1a; NM_131263). The real-time PCR procedure was conducted as previously described (38, 47). Serial dilutions were prepared from a pituitary cDNA sample, and the efficiencies of specific gene amplifications were compared by plotting Ct versus log (template). The PCR mixture contained 3 μl of a diluted cDNA sample, 400 nM of each primer, and 10 μl of Platinum SYBR Green qPCR SuperMix-UDG (Invitrogen) in a final volume of 20 μl. Amplification was carried out in a LightCycler96™ Real-Time PCR System (Roche) according to the manufacturer’s protocol. The cDNAs of the genes were amplified simultaneously in separate wells in duplicates, and the results were analyzed with the LightCycler96 software. A dissociation-curve analysis was conducted after each real-time experiment to confirm the presence of only one product. To eliminate false positives, a reverse-transcriptase negative control (RT-PCR) was run for each template and primer pair. Primer pair sequences and their slope and R^2^ values are listed in **Table 1**.

**Table 1:** Primers for real-time PCR.

Tissue	Primer	5’ to 3′ sequence	Slope	R2
	zfef1a-754F	ctagccgtcccaccgacaag	-3.46	0.99
	zfef1a-1151F	gcaggcgatgtgagcagtgt		
Brain	zfgnrh2-39F	gctgatgctgtgtctgagt	-3.06	0.99
	zfgnrh2-196R	tgtcttgaggatgtttcttc		
	zfgnrh3-149F	tggaggcaacattcaggatgt	-3.64	0.98
	zfgnrh3-253R	ccacctcattcactatgtgtatt		
	zfsst1.2-119F	ttgtgctatgtggtcgcagt	-3.36	0.99
	zfsst1.2-288R	ctcacgcatctgcatttcgg		
	zfkiss1-10F	acagacactcgtcccacagatg	-3.53	0.98
	zfkiss1-210R	caatcgtgtgagcatgtcctg		
	zfnpy-117F	ctgcttggggactctcacag	-3.95	0.99
	zfnpy-270R	gtcagcgcttgaccttttcc		
	zfar-1680F	atacggccgaagtactgctc	-2.94	0.87
	zfar-1855R	cactcctccctccgtcaaac		
	zfgper1-189F	tgcaggcggtgattcttgtt	-2.77	0.95
	zfgper1-351R	agtccagcgcagtcttgttt		
Pituitary	tigh-151F	tgctcgcccagagactcttc		
	tigh-351R	tgggaaactcccaggactca	-3.37	0.99
	zfgh-233F	cgccgacgggaaaagatgaa	-3.40	1.00
	zfgh-414R	atcccttgatgagcacgctg		
	Zflhb-190F	aatgcctggtgtttcagacc	-3.33	1.00
	zflhb-333R	aacagtcgggcaggttaatg		
	zfPrl-244F	caagccatgaaagtgccgga	-3.42	1.00
	zfprl-465R	gttgtcagaagacgagcccat		
	zftsh-39F	aatgaaggttgccgtgccta	-3.13	1.00
	zftsh-276R	aggatctgcatgtgaagggc		
	zfFsh-140F	Tgtgggagctgcgtcacaat	-3.53	1.00
	zffsh-327R	gccacggggtacacgaagac		
Liver	zfIgf1-127F	ggggcattggtgtgatgtct	-3.90	1.00
	Zfigf1-294R	ccagtgagagggtgtgggta		

Slope and R^3^ values were obtained from the validation assays of target and reference genes, with serially diluted cDNA, for the gene-specific primers used for the real-time PCR assays.

### Gonad histology

Gonadal histology was performed generally according to Mizrahi et al., 2019 (45). Images of histological slides of female gonads were analyzed using ImageJ FIJI software to determine follicle diameter. Imaged slides of male gonads were analyzed using CPCe 4.1 software to randomly assign 70 points, classify cell stage in spermatogenesis, and quantify stage frequencies according to Mizrahi et al., 2019 (45).

### Reproductive performances and fertility

Female reproductive performance was evaluated generally according to the formula RF=eggs (n)/body weight (mg). The relative fecundity, i.e., the ratio between the number of eggs spawned and female body weight, was calculated. Four-month-old sibling females of both lines were housed together. Mating of one female with one WT male was allowed for 1 hour. The female’s genotype was verified using the EGFP heart marker. Upon successful mating, we collected and counted the eggs, weighed the females, and housed them in a different tank. Following unsuccessful mattings, females were returned to the communal tank and were mated again on a different day.

Male fertility was assessed by measuring sperm concentration (48). Males were separated from females for 3 days before sperm collection. The night before sperm collection, males were housed with females in a mating tank, separated by a net. In the morning, male fish were anesthetized in tricaine and gently dried to remove excess water on the papilla. Sperm was collected immediately by placing a microcapillary tube on the papilla and carefully pressing the fish’s abdomen. The volume of extracted semen was calculated using a reference capillary, which contained a known volume of water. Sperm samples that contained urine were discarded. The sperm count was determined using a hemocytometer.

### Statistical analyses

Data are presented as mean ± SEM. Gaussian distribution tests (Kolmogorov–Smirnov and Shapiro–Wilk test) and unequal variance test (*F*-test) were performed with GraphPad Prism 8.0 software. Non-normally distributed data were analyzed using the non-parametric Mann-Whitney *U* test. Welch’s *t*-test was used to evaluate the significance of differences between group means when sample variances were not equal, but data distribution was normal. When sample variances were equal, a *t*-test was used. Statistical significance was defined as P < 0.05.

## Results

### Verification of transgene expression

To overcome the limitations of current fast-growing transgenic fish, particularly those related to abnormal morphology, we have developed a transgenic zebrafish expressing tilapia GH under the tilapia LH promoter (LHp-GH), that was previously tested (40). We chose the LH promoter rather than the FSH one since, for both tilapia and zebrafish, we found before a higher abundance of LH cells in the pituitary compared to FSH (40, 49). Additionally, our observations suggest that LH pituitary content exceeds that of FSH by 1-2 orders of magnitude (46, 50). This trend was reaffirmed in the current study, where the average LH content in wild-type zebrafish pituitaries was 10 times higher than FSH within the same group.

**Figure 1:**
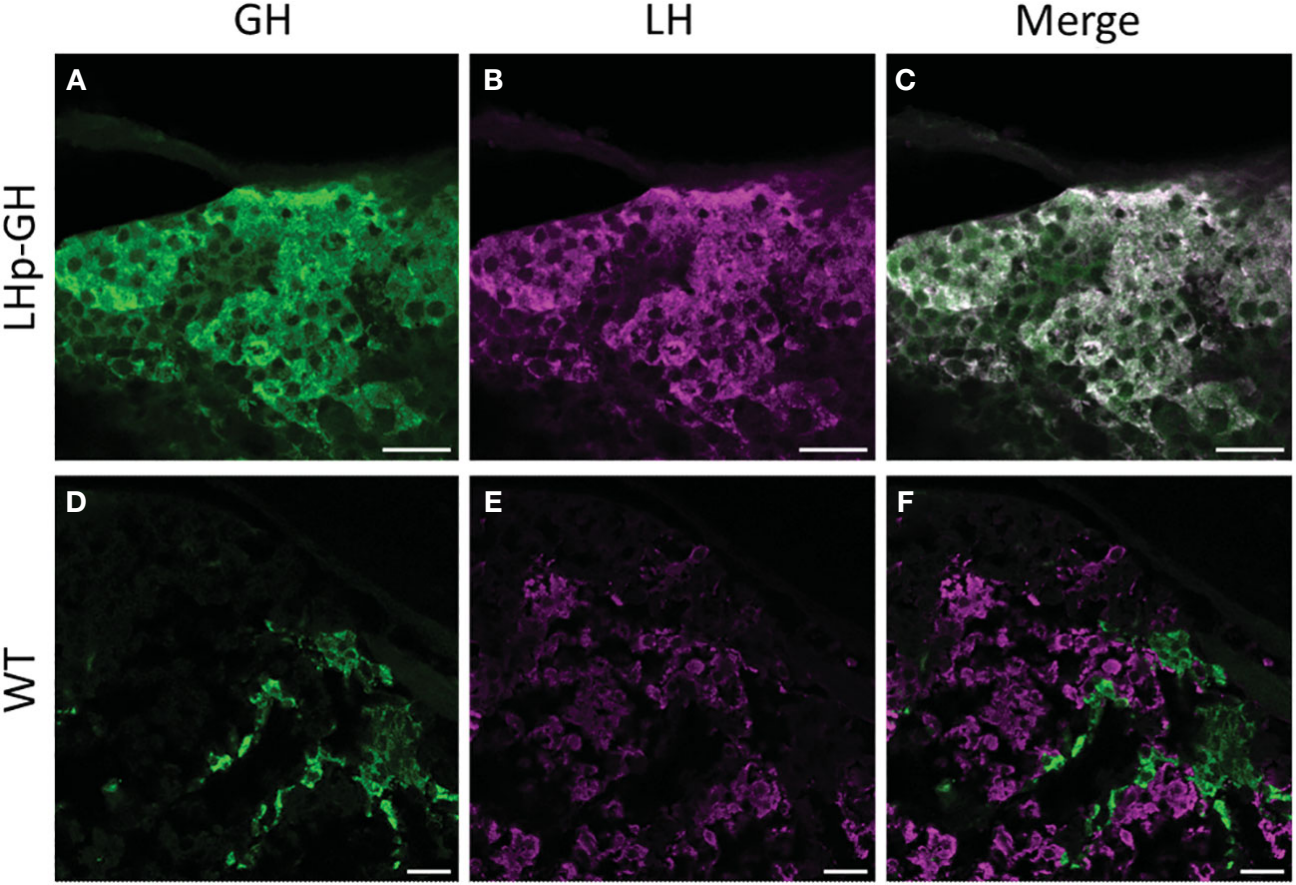
Distribution of LH and GH in the pituitary of zebrafish Tg(tiLHp:tiGH, cmlc2:EGFP) and WT. Confocal microscopy images of immunohistochemical staining of **(A, D)** GH (green); **(B,E)** LH (magenta); **(C)** merge of **(A, B)**; **(F)** merge of **(D, E)**. **(A–C)** Tg(LHp-tiGH); **(D–F)** WT. Scale bar = 20 µm.

To verify the presence of GH in LH cells in the pituitary of the transgenic fish, we performed immunohistochemistry analysis on pituitaries from LHp-GH and WT fish using specific and validated antibodies against GH and LH (**Figure 1**). Results showed an overlap between cells that synthesized GH (stained in green) and those that synthesized LH (stained in magenta) in LHp-GH fish pituitaries. By contrast, the two hormones were detected in different pituitary areas in WT fish.

### Growth performances

**Figure 2:**
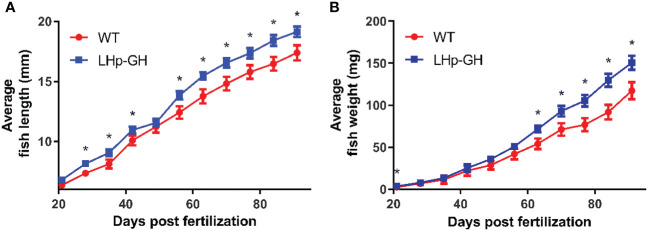
Accelerated weight and length gain in LHp-GH compared to WT fish. Weekly length **(A)** and weight **(B)** gain of LHp-GH fish (blue) and wild type (WT; red) fish. Fish were grown for 3 months and fed in excess (food weight for day >3% averaged body weight). n ranged between 85 to 55, depending on the week. The results are combined data from two repeats of the experiment. Asterisks indicate significant differences (P-value < 0.05).

To determine the effect of the transgene on growth, we compared length and weight between LHp-GH and WT fish. From 28 dpf onwards, the mean length of LHp-GH fish was significantly greater than that of WT fish, with the exception of a single measurement at 49 dpf, when the mean length of the LHp-GH fish was higher but not statistically significant (**Figure 2A**; *t*-test). Likewise, LHp-GH fish were significantly heavier than the WT fish at 21 dpf and from 63 dpf until the end of the experiment (**Figure 2B**).

**Figure 3:**
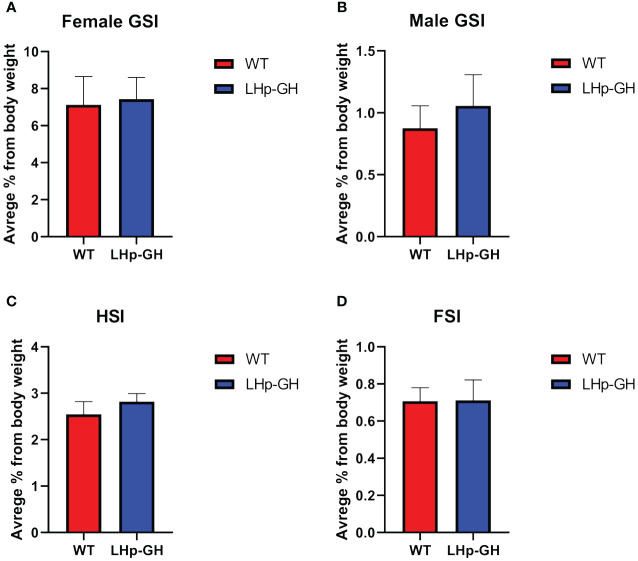
Comparisons of somatic indices between genotypes. Graph showing somatic indices in LHp-GH and WT fish. **(A)** GSI comparison in females (LHp-GH, n=14; WT, n=8). **(B)** GSI comparison in males (LHp-GH, n=15; WT, n=7). **(C)** HSI comparison between lines (LHp-GH, n=30; WT, n=19). **(D)** FSI comparison between lines (LHp-GH, n=23; WT, n=12). Values represent the mean ± SEM.

To evaluate the effects of the transgene on fish physiology, we calculated several somatic indices. The GSI is a well-established indicator of reproductive activity and sexual maturation, the HSI informs on the level of vitellogenin synthesis in the liver, and the visceral FSI reveals the accumulation and degradation of fat tissue, reflecting metabolic function. The results showed no significant difference between LHp-GH and WT fish in any index (**Figure 3**). These findings show that the increase in body mass did not derive from changes in gonadal, hepatic, or fat tissue mass and is more likely to result from an increase in muscle mass, bone mass or both. Examination of gross morphology showed no apparent external changes in body proportion or deformities in LHp-GH fish compared to WT fish.

**Figure 4:**
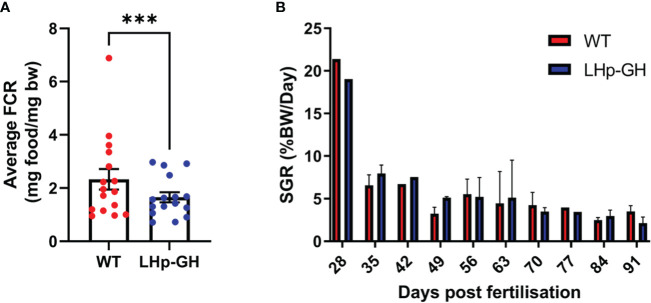
Comparison of FCR and SGR between LHp-GH and WT fish. **(A)** Teekly FCR values of the fish lines. The weekly FCR values from both experiments are matched and compared (Wilcoxon matched-pairs test; whiskers represent SEM; n=16 (Three asterisks indicate p-value < 0.001). **(B)** Dfferences in the mean weekly SGR for each week of the experiment (n=4) (Wilcoxon matched-pairs test; whiskers represent SD).

Next, to assess growth in the LHp-GH fish compared to the WT, we calculated FCR and SGR values based on the mean weight of the fish as measured weekly. Examination showed a significant difference in weekly FCR values between the fish lines (**Figure 4A**, *p*-value ≤ 0.0005, Wilcoxon matched-pairs test). LHp-GH fish weekly FCR values averaged 1.65, while WT fish averaged 2.32. we were not able to detect significant changes between the weekly SGR values of the two lines (**Figure 4B**).

### Gonadotropins and growth hormone content in pituitaries of LHp-GH and WT fish

**Figure 5:**
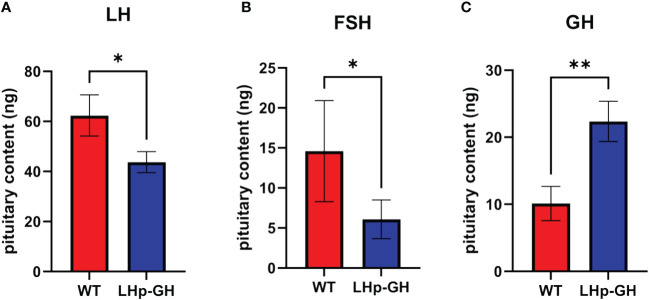
GtH and GH pituitary content in LHp-GH and WT zebrafish. **(A)** LH content in pituitaries of LHp-GH and WT fish; **(B)** FSH content in pituitaries of LHp-GH and WT fish; **(C)** GH content in pituitaries of LHp-GH and WT fish; for LHp-GH fish n=13, for WT fish n=6. Data are presented as mean ± SEM; Asterisks indicate p-value<0.05, double asterisks indicate p-value<0.01.

To determine the effect of the transgene on the main hormones related to reproduction and growth, we measured zebrafish LH and FSH and tilapia GH levels in the pituitaries of LHp-GH and WT fish. The results showed significantly higher LH and FSH pituitary content in WT fish, while significantly higher levels of GH were measured in LHp-GH fish (**Figure 5**; Mann-Whitney *U*-test). Comparison between sexes showed that LH content was higher in females than in males, whereas FSH and GH levels were similar in males and females (**Supplementary Figure 2**).

### Expression analysis of growth and reproduction genes

**Figure 6:**
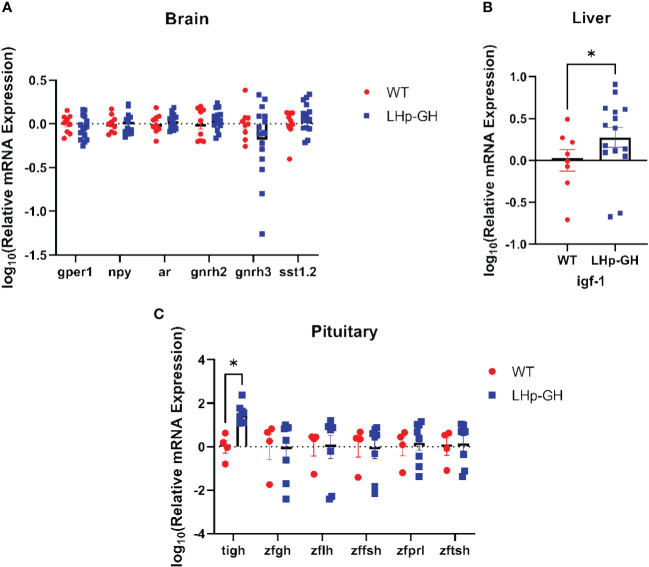
Comparison of mRNA expression of growth and reproduction genes. Expression levels of various genes as measured by real-time RT-PCR analysis in the brain **(A)**, liver **(B)** and pituitary **(C)** of LHp-GH and WT fish. Levels of expression were normalized to that of the housekeeping gene ef1a. Statistical significance was measured using two-way ANOVA (brain: LHp-GH fish n=16, WT fish n=9; pituitary: LHp-GH fish n=8, WT fish n=4; liver: LHp-GH fish n=15, WT fish n=8); asterisks indicate significant differences.

Next, we tested the mRNA levels of essential genes along the growth and reproduction axes. In the brain we tested for: G protein-coupled estrogen receptor 1 (gper1, NM_001128723.1), neuropeptide Y (npy, NM_131074.2), androgen receptor (ar, NM_001083123.1), gonadotropin-releasing hormone 2 (gnrh2, NM_181439.4), gonadotropin-releasing hormone 3 (gnrh3, NM_182887.2), and somatostatin 1, tandem duplicate 2 (NM_001386222.1). in the pituitary we tested for: tilapia growth hormone 1 (tigh, XM_003442542.5), zebrafish growth hormone 1 (zfgh, NM_001020492.2), zebrafish luteinizing hormone (zflh, NM_205622.2), zebrafish follicle stimulating hormone (zffsh NM_205624.1), zebrafish prolactin (zfprl, NM_181437.3), and zebrafish thyroid stimulation hormone (zftsh, NM_181494.2). in the liver we tested for insulin-like growth factor 1 (ifg-1, NM_131825.2). RT-PCR analysis of fish brains showed no significant difference in mRNA levels between LHp-GH and WT fish for any of the examined genes (**Figure 6A**; Mann-Whitney test). As expected, LHp-GH fish displayed significantly higher tiGH in the pituitary, further validating the construct activity (**Figure 6C**; Mann-Whitney test). No other examined gene differed significantly in pituitary expression between the fish lines. However, IGF-1 levels in the liver were significantly higher in LHp-GH than WT fish (**Figure 6B**).

### Follicle maturation and frequency of spermatogenesis stages

**Figure 7:**
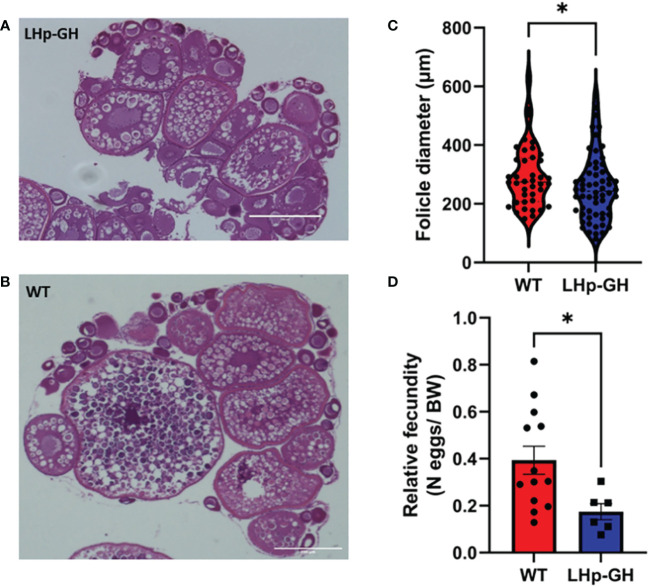
Gonad follicle size and relative fecundity in female LHp-GH vs. WT fish. Examples of stained and imaged gonadal sections from female LHp-GH **(A)** or WT fish **(B)**; scale bars, 400 µm. Mean follicle diameter of the 10 largest follicles in each slide in the gonads of female LHp-GH and WT fish **(C)**. Mean relative fecundity of female LHp-GH and WT fish **(D)**. (LHp-GH; n=60, WT; n=40; t-test). Data are presented as mean ± SEM. Asterisks in **(C, D)** indicate significant differences.

To determine the effect of elevated GH levels on the development of gonads, we performed image analysis on histological sections of ovaries and testes (**Figures 7A, B**). Because female zebrafish are batch spawners and spawn throughout the year, they constantly carry follicles at various stages of maturation. Accordingly, the mean follicle diameter is highly varied and could not be used to measure follicle maturation. Instead, we calculated the mean of the 10 largest follicle diameters closest to ovulation, thus reducing the variation considerably. The results showed that the mean diameter of these follicles in the gonad was significantly lower in LHp-GH fish than in WT fish (**Figure 7C**; Mann-Whitney test). We saw a similar trend in relative fecundity, as LHp-GH females spawned fewer eggs relative to their body weight (**Figure 7D**; *t*-test). In males, neither the distribution of spermatogenesis stages nor sperm concentrations differed significantly between the fish lines (**Supplementary Figure 3**).

## Discussion

In vertebrates, the somatotropic axis constantly interacts with other physiological pathways that control energy homeostasis, metabolism and reproduction to regulate somatic growth. Thus, transgenic fish that produce high levels of GH have immense potential for science, medicine, and aquaculture. Indeed, transgenic GH lines of various teleosts generated in the last decades display accelerated growth. However, in most cases, a highly active promoter was used to drive the expression of GH, resulting in fish that are more prone to health problems and severe morphological abnormalities (22–27). In this instance, we utilized an alternative GH transgenesis approach, incorporating a less active and, more precisely, localized LH promoter. Upon confirming the construct’s efficacy by demonstrating the expression of exogenous GH in LH cells of the pituitaries in this new LHp-GH zebrafish, we conducted a more in-depth characterization of these novel fish lines. Our results show that they exhibited accelerated growth in length and weight, lower FCR values, and higher expression and production levels of tiGH in the pituitary and of IGF-1 expression in the liver, combined with reduced GtH production levels. Analysis of reproductive parameters unveiled that female LHp-GH fish exhibited smaller gonad follicles and a reduced egg-spawning count compared to weight-matched WT females. This suggests a resource redirection from reproduction to somatic growth in the LHp-GH fish.

Growth performance is of the utmost importance for aquaculture. In the last decades, many newly developed lines of GH transgenic fish from a variety of species, including coho salmon (22), domestic and wild rainbow trout (23), catfish (24), common carp (25), tilapia (26), and zebrafish (27), have displayed intensified growth, as they were heavier and longer than their WT counterparts. The findings of our study are consistent with those previous results. The mean length of the LHp-GH fish was significantly higher throughout most of the experiment. Body weights also differed significantly from the second half of the experiment since LHp-GH fish were heavier than WT fish.

The FCR parameter represents the efficiency with which an organism converts food into body mass, a process that is tightly regulated by the somatotropic axis. The Feed Conversion Ratio (FCR) values in fish bear significant implications for aquaculture, with GH transgenic fish frequently demonstrating lower values. This observation suggests that GH-transgenic fish tend to achieve greater body mass than wild-type (WT) counterparts while consuming an equivalent amount of food (51, 52). Our results show a similar trend, as LHp-GH fish displayed lower FCR values. Combined with the increases in length and weight, these findings strengthen the conclusion that LHp-GH fish devote more energy and resources to somatic growth.

Interestingly, some of our results were inconsistent with those of former studies. For instance, the SGR did not differ between LHp-GH and WT fish. Previous studies measuring SGR in transgenic GH fish reported significantly higher values than WT fish. This was shown in coho salmon in which metallothionein-b promotor drove GH expression (22, 23), in common carp in which grass carp GH driven by β-actin gene promoter of common carp was used (25), and in Nile Tilapia where the medaka β-actin promoter drove tiGH expression (53). One reason for this inconsistency may be the use of different statistical analyses. We calculated SGR values for each week separately and then compared these values between LHp-GH and WT fish by using matched pairs for all weeks, whereas other studies have usually employed a *t*-test or Mann-Whitney u-test on SGR data from a single period to compare multiple groups or individuals of each treatment (54).

During the experiment, while no significant differences in SGR were observed between the two fish lines, significant differences in length and weight were noted. However, it is possible that there were some specific periods of SGR variation that went undetected. Such transient fluctuations could have contributed to minor disparities in weight, which subsequently amplified throughout the study. It’s important to note that variations in weight can manifest even when SGR values are comparable. This phenomenon arises because expressing growth as a percentage of body weight results in different weight gains among subjects with varying initial body weights.

Somatic indexes, such as the GSI, HSI, and VFSI, provide information about physiological processes such as fat accumulation, gonadal development, environmental adaptation, and general well-being (55). We found no significant change in GSI, HSI, or VFSI between LHp-GH and WT fish. Similar GSI values suggest that reproductive development is strongly coupled with body weight, such that the high GH levels did not direct energy and resources toward somatic growth at the expense of sexual development. Bessey et al. (54) reported similar findings and conclusions after identifying an unchanged GSI in GH transgenic coho salmon (56). However, the decrease in FSH and LH pituitary content and follicle diameter may suggest that such redirection of energy does occur, as has been shown in other studies on fish. For example, it has been shown that common carp that overexpress GH exhibit a reduction in GSI compared to their WT counterparts (57–59). Furthermore, in GH transgenic tilapia, it was observed that the female GSI was lower in transgenic individuals compared to their non-transgenic siblings in both mixed and separate culture conditions. Additionally, transgenic male GSI values were lower in separate cultures than their non-transgenic siblings (60).

In any case, the unchanged somatic indices could imply that the bodies of the LHp-GH fish remained proportional and similar to those of the WT fish and that the weight gain that the LHp-GH fish exhibit is not the result of increment in gonadal, hepatic or fat tissues. If so, it is possible that the conservation of body proportion in our LHp-GH fish may result from lower and localized GH expression, as compared to other GH transgenic fish which might allow the LHp-GH fish to retain their enhanced growth while avoiding body deformation. The unchanged body proportions could be beneficial in aquaculture. In some transgenic fish lines, overexpression of GH under the regulation of a highly active promoter, such as β-actin or metallothionein-B, has caused unwanted deformities, such as acromegaly, abnormal body shape, and changes in organ locations (23, 28–30). These morphological changes might affect fish health and consumer acceptance. Thus, using the LHp : GH construct, which ended with increased fish size while preserving their body proportion, produces more suitable fish for consumption.

Inregard to gonadotropin levels in the pituitary, our findings indicate a notable decrease in LH and FSH pituitary content in LHp-GH fish compared to WT fish. This finding is consistent with the findings of (61) who reported GH inhibition upon LH release from neighboring GH cells in common carp, in addition, elevated igf-1 levels have been shown to increase LH content in the pituitary (16, 62). These two interactions act apparently in opposition to each other. However, in the current study we observed a decrease in gonadotropin levels, indicating that the regulatory effect of GH overexpression on the gonadotropins was more pronounced than the effects of elevated igf-1. Consequently, we can suggest that the observed changes in reproductive parameters were caused by high GH, levels that inhibits the release of LH and FSH.

Concerning gonad morphology, we found that LHp-GH females have smaller follicles than WT females. These results are consistent with previous findings in salmonids (56, 63) Yet, whereas Bessey et al. (54) reported decreased egg diameter but increased egg number in GH transgenic coho salmon, which led to unchanged GSIs, Jonsson et al. (61) showed that Atlantic salmon displayed a phenotypically plastic response to reduction in juvenile resource abundance and growth opportunities by increasing egg size and decreasing fecundity (56, 63). The current study demonstrated a different trend, as both relative fecundity and follicle size decreased. This implies that the alterations in the reproductive parameters of female tilapia may not solely be attributed to elevated GH levels but may also result from the integration of GH with reproductive processes.

Our findings related to endocrine regulation may offer a possible explanation for the observed morphological changes. As expected, gene expression analysis revealed gene changes associated with the somatotropic axis and growth regulation. tiGH expression level was significantly elevated in the pituitary, alongside IGF-1 level in the liver. These two hormones are critical components in the somatotropic axis, and therefore, their upregulation may account for the changes noted in both growth and feed efficiency (1).

Another intriguing revelation from these findings is the interaction between tilapia GH and the zebrafish growth hormone receptor (zfGHr). Considering the considerable phylogenetic distance between these two fish species in the piscine world, it was noteworthy to discover that tiGH could bind to the zfGHr and induce effects such as increased IGF-1 expression levels and growth. These effects likely contributed to the overall enhanced growth performances that were consistently observed throughout the study. Although the exact affinity of zfGHr for tiGH and the efficiency of this binding in mediating effects in target cells are yet to be determined, we can confidently posit that tiGH is indeed capable of binding to zfGHr.

In summary, our primary observations highlight that the LHp-GH zebrafish line demonstrated accelerated growth and more efficient conversion of food into body mass, likely attributed to the heightened activation of the somatic axis. Despite the rapid growth, the preservation of body proportions suggests the viability of employing this transgenic construct in genetic engineering for commercial aquaculture fish species. Furthermore, we suggest that the decline in reproductive parameters results from the co-regulation of GH and LH expression, promoting the redirection of metabolic resources from reproduction to somatic growth. With the exception of fish species that are grown for their gonads or eggs, this shift is a highly desirable trait that further qualifies the LHp : GH construct as a highly potential candidate for effective GH transgenesis.

## Data availability statement

The raw data supporting the conclusions of this article will be made available by the authors, without undue reservation.

## Ethics statement

The animal study was approved by Animal Care and Use Guidelines of the Hebrew University and were approved by the local Administrative Panel on Laboratory Animal Care. The study was conducted in accordance with the local legislation and institutional requirements.

## Author contributions

NC-R: Data curation, Formal analysis, Investigation, Methodology, Writing – original draft. NM: Conceptualization, Investigation, Writing – review & editing. BL-S: Conceptualization, Funding acquisition, Resources, Supervision, Writing – review & editing.
