# Supplementary material for: Characterization of a novel fast-growing zebrafish: a new approach to growth hormone transgenesis
Source: Front Endocrinol (Lausanne). 2024 Apr 2;15:1369043. doi: 10.3389/fendo.2024.1369043 (PMC11018968; doi:10.3389/fendo.2024.1369043)
Supplement: Supplementary file 1 [file DataSheet_1.pdf]

Cohen-Rothschild et al.,
Supplementary data

Validation of the ELISA developed for cFSH and cLH for zebrafish.

Although the ELISA for the quantitative measurement of cFSH and cLH had already been firmly established in the lab (Aizen et al., 2017; Hollander-Cohen et al., 2018), it had not been fully calibrated for zebrafish pituitary content. To this end, the first step was to determine the exact pituitary dilution to adopt in each ELISA to retrieve reliable results. For the ELISA of FSH measurements, the standard curve ranged from 508 fg/ml to 10 ng/ml. For the ELISA of LH measurements, the curve ranged from 10.67 pg/ml to 210 ng/ml. The R^2 values for the standard curves were 0.9613 for cFSH and 0.9379 for cLH, while the sensitivity (lower limit of detection) was 3 pg/ml for cFSH and 58 pg/ml for cLH.

Ensuring parallelism is important when validating an assay for application with biological samples. In this process, dilutions of the sample are graphed against the standard curve and subsequently tested. The lines generated through serial dilutions of the standard and biological samples (initial dilution: 1 pituitary in 100 µL, dilution factor of 1:5) exhibit parallel lines. Notably, the slopes of these lines show no significant differences (FSH: P=0.297; LH: P=0.698). As a result, we can conclude that the current assay enables us to accurately determine FSH and LH levels in zebrafish pituitary tissues using antibodies that were developed against carp FSH and LH.

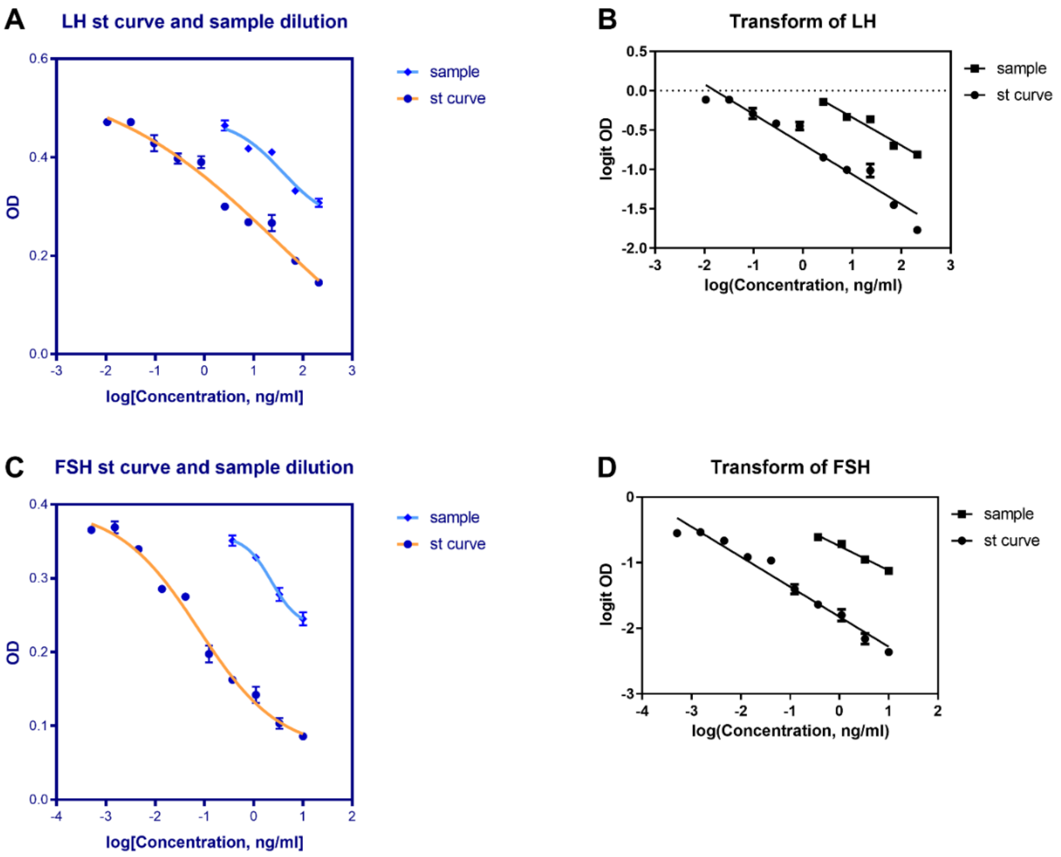

Supplementary Figure 1: Parallelism of recombinant carp gonadotropins and native zebrafish pituitary extract

Comparison of the standard curves of the ELISA for carp LH (A, B) and FSH (C, D) with those of zebrafish pituitary extract (ZPE).

Differences in LH, FSH and GH content between males and females

we noted that the LH content was higher in females than males, while the FSH levels were similar between males and females (Figure S2).

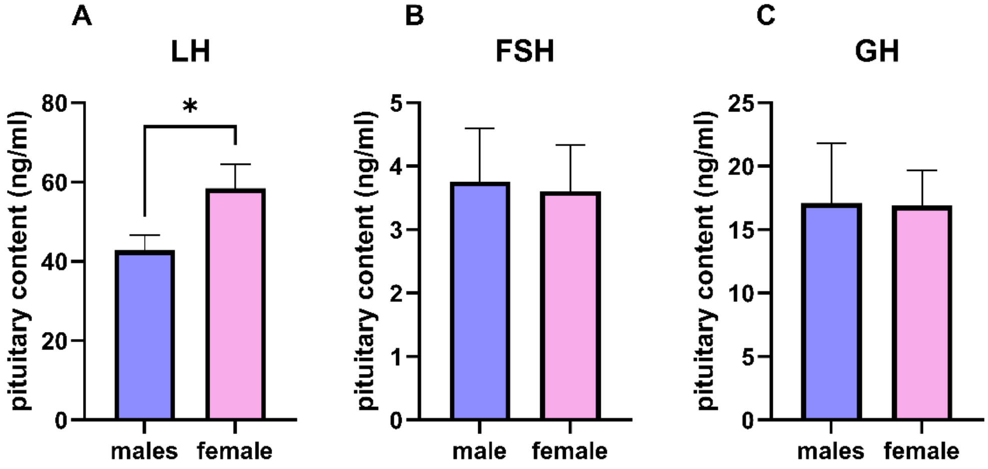

Supplementary Figure 2: Zebrafish GtH and GH pituitary content in the LHp-GH and WT female and male fish

represented by mean ± SEM; Male fish n=6, Female fish n=11. Asterisks indicate significant differences.

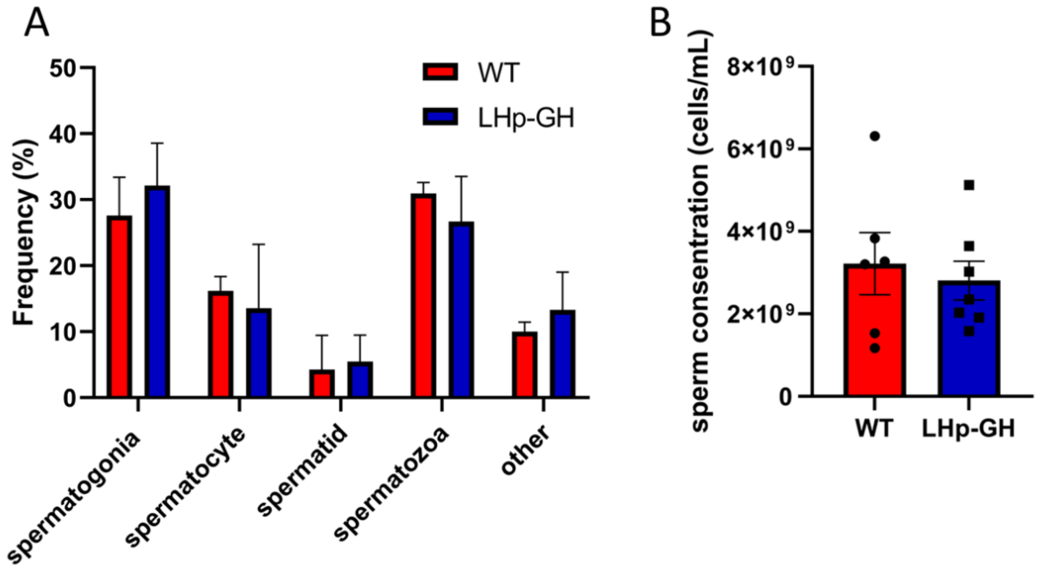

Supplementary Figure 3: male reproductive performances.
A) A comparison of the frequency of different stages in spermatogenesis between LHp-GH and WT zebrafish. A chi-square statistical test was used. The bars and whiskers represent the mean value and SEM, respectively. **B)** sperm count in LHp-GH and WT zebrafish. A one-tailed non-parametric Mann-Whitney test was used. The bars and whiskers represent the mean value and SEM, respectively.
